# Serious Adverse Health Events, Including Death, Associated with Ingesting Alcohol-Based Hand Sanitizers Containing Methanol — Arizona and New Mexico, May–June 2020

**DOI:** 10.15585/mmwr.mm6932e1

**Published:** 2020-08-14

**Authors:** Luke Yip, Danae Bixler, Daniel E. Brooks, Kevin R. Clarke, S. Deblina Datta, Steven Dudley, Kenneth K. Komatsu, Jennifer N. Lind, Annaliese Mayette, Michael Melgar, Talia Pindyck, Kristine M. Schmit, Steven A. Seifert, Farshad Mazda Shirazi, Susan C. Smolinske, Brandon J. Warrick, Arthur Chang

**Affiliations:** ^1^CDC COVID-19 Response Team; ^2^Banner Poison and Drug Information Center, Phoenix, Arizona; ^3^Arizona Poison and Drug Information Center, Tucson, Arizona; ^4^Division of Public Health Preparedness, Arizona Department of Health Services; ^5^Epidemiology and Response Division, New Mexico Department of Health; ^6^University of New Mexico School of Medicine, Albuquerque, New Mexico; ^7^New Mexico Poison and Drug Information Center, Albuquerque, New Mexico.

Alcohol-based hand sanitizer is a liquid, gel, or foam that contains ethanol or isopropanol used to disinfect hands. Hand hygiene is an important component of the U.S. response to the emergence of SARS-CoV-2, the virus that causes coronavirus disease 2019 (COVID-19). If soap and water are not readily available, CDC recommends the use of alcohol-based hand sanitizer products that contain at least 60% ethyl alcohol (ethanol) or 70% isopropyl alcohol (isopropanol) in community settings (*1*); in health care settings, CDC recommendations specify that alcohol-based hand sanitizer products should contain 60%–95% alcohol (≥60% ethanol or ≥70% isopropanol) (*2*). According to the Food and Drug Administration (FDA), which regulates alcohol-based hand sanitizers as an over-the-counter drug, methanol (methyl alcohol) is not an acceptable ingredient. Cases of ethanol toxicity following ingestion of alcohol-based hand sanitizer products have been reported in persons with alcohol use disorder (*3*,*4*). On June 30, 2020, CDC received notification from public health partners in Arizona and New Mexico of cases of methanol poisoning associated with ingestion of alcohol-based hand sanitizers. The case reports followed an FDA consumer alert issued on June 19, 2020, warning about specific hand sanitizers that contain methanol. Whereas early clinical effects of methanol and ethanol poisoning are similar (e.g., headache, blurred vision, nausea, vomiting, abdominal pain, loss of coordination, and decreased level of consciousness), persons with methanol poisoning might develop severe anion-gap metabolic acidosis, seizures, and blindness. If left untreated methanol poisoning can be fatal (*5*). Survivors of methanol poisoning might have permanent visual impairment, including complete vision loss; data suggest that vision loss results from the direct toxic effect of formate, a toxic anion metabolite of methanol, on the optic nerve (*6*). CDC and state partners established a case definition of alcohol-based hand sanitizer–associated methanol poisoning and reviewed 62 poison center call records from May 1 through June 30, 2020, to characterize reported cases. Medical records were reviewed to abstract details missing from poison center call records. During this period, 15 adult patients met the case definition, including persons who were American Indian/Alaska Native (AI/AN). All had ingested an alcohol-based hand sanitizer and were subsequently admitted to a hospital. Four patients died and three were discharged with vision impairment. Persons should never ingest alcohol-based hand sanitizer, avoid use of specific imported products found to contain methanol, and continue to monitor FDA guidance (*7*). Clinicians should maintain a high index of suspicion for methanol poisoning when evaluating adult or pediatric patients with reported swallowing of an alcohol-based hand sanitizer product or with symptoms, signs, and laboratory findings (e.g., elevated anion-gap metabolic acidosis) compatible with methanol poisoning. Treatment of methanol poisoning includes supportive care, correction of acidosis, administration of an alcohol dehydrogenase inhibitor (e.g., fomepizole), and frequently, hemodialysis.

A case of alcohol-based hand sanitizer–associated methanol poisoning was defined as detectable blood methanol concentration and a history of alcohol-based hand sanitizer exposure (e.g., ingestion, dermal, ocular, inhalation, or injection) in any person who sought medical attention in Arizona or New Mexico during May 1–June 30, 2020. To identify and characterize cases, CDC collaborated with Arizona Department of Health Services, Arizona Poison and Drug Information Center System, New Mexico Department of Health, and New Mexico Poison and Drug Information Center to identify and review poison center call records. Clinical and demographic data were abstracted from records that met the case definition. Patients were characterized according to age, sex, signs and symptoms at evaluation, blood test results, including methanol levels, presence of anion-gap acidosis, treatments received, and outcomes. Medical records were reviewed for clinical and demographic details missing from the poison center call records. An illustrative case vignette is presented. Activity was determined to meet the requirements of public health surveillance as defined in 45 CFR 46.102(l)(2).

During May 1–June 30, 15 cases of alcohol-based hand sanitizer–associated methanol poisoning were identified, including persons who were AI/AN (Table). All patients had reportedly ingested hand sanitizer, and all were admitted to a hospital. The mean patient age was 43 years (range = 21–65 years); 13 were male. All patients had a history of swallowing alcohol-based hand sanitizer products. The earliest available blood methanol concentrations ranged from 21 mg/dL to >500 mg/dL. All patients had evidence of a metabolic acidosis: anion gap levels ranged from 17 to 49 milliequivalents per liter (mEq/L) (normal = 3–10), serum bicarbonate concentrations ranged from <5 to 13 mEq/L (normal = 22–28), and blood pH ranged from 6.70 to 7.25 (normal = 7.35–7.45). Six patients developed seizures during their hospitalization. All patients were treated with fomepizole (a competitive inhibitor of alcohol dehydrogenase, the enzyme that catalyzes the initial step in the metabolism of methanol to its toxic metabolites), and nine received hemodialysis or continuous renal replacement therapy. As of July 8, four patients remain hospitalized. Among seven patients discharged from the hospital, four had no sequelae, and three were discharged with new visual impairment. Among the four patients who died, three had seizures at the time of admission; initial signs and symptoms were not reported for the fourth patient.

**TABLE:** Characteristics of patients admitted to health care facilities with methanol poisoning associated with ingestion of alcohol-based hand sanitizer products containing methanol — Arizona and New Mexico, May–June 2020

Age (yrs)	Sex	Chief complaint(s)*	Serum methanol concentration (mg/dL)	Anion gap^†^ (mEq/L)	Serum bicarbonate^§^ concentration (mEq/L)	Blood pH^¶^	Treatment	Outcome
21	M	Gastrointestinal	44	30	6	7.15	4MP	D/C, no sequelae
30	M	Visual disturbance	35	43	11	N/A	4MP	D/C, no sequelae
35	M	Unresponsive, seizures	198	49	<5	6.87	4MP	Died
36	M	Decreased responsiveness	>500	42	7	7.23	4MP, HD	Remains hospitalized**
38	M	Gastrointestinal	131	35	<5	6.81	4MP, HD, CRRT	D/C, no sequelae
38	F	N/A	21^††^	N/A	N/A	N/A	4MP	Died
39	M	Seizures, unconscious	278	23	11	N/A	4MP, HD	Died
40	M	Dog bite	319	35	<5	7.00	4MP, CRRT	Remains hospitalized**
44	M	Visual disturbance, seizures	97	32	<6	7.09	4MP, HD	D/C with visual impairment
47	M	Headache, visual disturbance	43	34	8	7.25	4MP, HD	D/C with visual impairment
50	M	Visual disturbance	410	22	9	6.70	4MP, CRRT	Remains hospitalized**
51	F	Dyspnea	42	23	6.2	7.14	4MP	D/C with visual impairment
54	M	Media alert^§§^	56	17	13	N/A	4MP	D/C, no sequelae
63	M	Altered mental status	548	30	11	7.12	4MP, HD	Remains hospitalized**
65	M	Unresponsive, seizures, cardiac arrest	308	31	<5	N/A	4MP, HD, CRRT	Died

**Abbreviations:** CRRT = continuous renal replacement therapy; D/C = discharged from hospital; F = female; HD = hemodialysis; M = male; mEq = milliequivalents; 4MP = fomepizole; N/A = not available.

* Chief complaint(s) directly came from medical records. Laboratory data were earliest recorded results.

^†^ Normal = 3–10 mEq/L; elevated levels can indicate metabolic acidosis.

^§^ Normal = 22–28 mEq/L.

^¶^ Normal = 7.35–7.45.

** As of July 8, 2020.

^††^ 2 days after admission.

^§§^ Patient saw media report on alcohol-based hand sanitizers containing methanol and wanted to be evaluated by a medical professional.

## Illustrative Case

A man aged 44 years was evaluated at a health care facility for recent onset of visual impairment. The patient reported drinking an unknown quantity of alcohol-based hand sanitizer during the few days before seeking medical care. Initial laboratory investigations were notable for a blood methanol concentration of 97 mg/dL and metabolic acidosis, with an anion gap of 32 mEq/L, serum bicarbonate concentration of <6 mEq/L, and arterial blood pH of 7.09. His clinical course was complicated by seizures. The patient was treated with fomepizole and underwent hemodialysis. He recovered after a 6-day hospitalization for acute methanol poisoning and was discharged with near-total vision loss.

## Discussion

In addition to social distancing and consistent use of face masks, hand hygiene is an integral component of the response to the emergence of SARS-CoV-2 in the United States.* Practicing hand hygiene, for example, by washing hands with soap and water for at least 20 seconds,^†^ is a simple and effective way to decrease the spread of pathogens and infections. If soap and water are not readily available, CDC recommends the use of alcohol-based hand sanitizer products that contain at least 60% alcohol (ethanol or isopropanol) in community settings (*1*); alcohol-based hand sanitizers used in health care settings should contain 60%–95% alcohol (≥60% ethanol or ≥70% isopropanol) (*2*). This investigation highlights the serious adverse health events, including death, that can occur after ingesting alcohol-based hand sanitizer products containing methanol. Safety messaging to avoid ingestion of any alcohol-based hand sanitizer product should continue.^§^ Similar cases of methanol toxicity might be occurring in other states and localities.

Swallowing alcohol-based hand sanitizer products containing methanol can cause life-threatening methanol poisoning. Young children might unintentionally swallow these products, whereas adolescents or adults with history of alcohol use disorder might intentionally swallow these products as an alcohol (ethanol) substitute (*3*,*4*). 

Although methanol can be absorbed through the skin (*8*), transcutaneous methanol poisoning is rare and has been reported under unusual circumstances (*9*). The extent and rate of transcutaneous methanol absorption depends on variables such as its form (e.g., vapors, liquid, or solution), contact time, dose, concentration, and size of the exposure area (*8*,*10*).^¶^

The findings in this report are subject to at least two limitations. First, the clinical diagnosis of methanol poisoning can be challenging because eliciting an exposure history can be challenging for patients with altered mental status, and some hospitals might be unable to test for a blood methanol level. Cases of methanol poisoning might not have been recognized or reported to poison centers or state health departments. Second, the extent of potential exposure to alcohol-based hand sanitizer products containing methanol is uncertain; additional cases might be identified. As of July 15, 2020, FDA had tested and identified 67 alcohol-based hand sanitizer products that contain methanol (*7*). These products are being recalled by the manufacturer or distributor in the United States. An FDA investigation is ongoing.

Severe methanol poisoning resulting in permanent disability or death can occur after swallowing alcohol-based hand sanitizer containing methanol. The public should check their products against the FDA Updates on Hand Sanitizers Consumers Should Not Use website (*7*). If the product is on this list, its use should be discontinued immediately, and the product should be disposed of in hazardous waste containers; these products should not be flushed down a toilet or poured down a drain.** All alcohol-based hand sanitizers should only be used to disinfect hands and should never be swallowed. Children using hand sanitizers should be supervised, and these products should be kept out of reach of children when not in use. Swallowing alcohol-based hand sanitizer products, including those that do not contain methanol, might also lead to serious illness and outcomes, including death (*3*,*4*). Consumers who have been exposed to alcohol-based hand sanitizers containing methanol should stop using them immediately and seek immediate medical attention if they experience any concerning symptoms. Clinicians should have a high index of suspicion for methanol poisoning when evaluating patients with either a history of swallowing an alcohol-based hand sanitizer or compatible signs and symptoms and, if needed, obtain medical management advice from their regional poison center (1-800-222-1222).

**Summary**

**What is already known about this topic?**

Alcohol-based hand sanitizers should only contain ethanol or isopropanol, but some products imported into the United States have been found to contain methanol.

**What is added by this report?**

From May 1 through June 30, 2020, 15 cases of methanol poisoning were reported in Arizona and New Mexico, associated with swallowing alcohol-based hand sanitizers. Four patients died, and three were discharged with visual impairment.

**What are the implications for public health practice?**

Alcohol-based hand sanitizer products should never be ingested. In patients with compatible signs and symptoms or after having swallowed hand sanitizer, prompt evaluation for methanol poisoning is required. Health departments in all states should coordinate with poison centers to identify cases of methanol poisoning.

## Footnotes

* https://www.cdc.gov/handwashing/when-how-handwashing.html.

† https://www.cdc.gov/coronavirus/2019-ncov/prevent-getting-sick/prevention.html.

§ https://www.cdc.gov/handwashing/show-me-the-science-hand-sanitizer.html.

¶ https://www.sciencedirect.com/science/article/pii/S0022202X1546837X.

**
https://www.fda.gov/drugs/drug-safety-and-availability/fda-advises-consumers-not-use-hand-sanitizer-products-manufactured-eskbiochem .
